# Potential Involvement of Peripheral Leptin/STAT3 Signaling in the Effects of Resveratrol and Its Metabolites on Reducing Body Fat Accumulation

**DOI:** 10.3390/nu10111757

**Published:** 2018-11-14

**Authors:** Andrea Ardid-Ruiz, Maria Ibars, Pedro Mena, Daniele Del Rio, Begoña Muguerza, Cinta Bladé, Lluís Arola, Gerard Aragonès, Manuel Suárez

**Affiliations:** 1 Department of Biochemistry and Biotechnology, Nutrigenomics Research Group, Universitat Rovira i Virgili, 43007 Tarragona, Spain; andrea.ardid@urv.cat (A.A.-R.); maria.ibars@urv.cat (M.I.); begona.muguerza@urv.cat (B.M.); mariacinta.blade@urv.cat (C.B.); lluis.arola@urv.cat (L.A.); manuel.suarez@urv.cat (M.S.); 2 Department of Food and Drugs, Human Nutrition Unit, University of Parma, 43125 Parma, Italy; pedromiguel.menaparreno@unipr.it; 3 Department of Veterinary Medicine, University of Parma, 43125 Parma, Italy; daniele.delrio@unipr.it; 4 School for Advanced Studies on Food and Nutrition, University of Parma, 43125 Parma, Italy; 5 Microbiome Research Hub, University of Parma, 43125 Parma, Italy; 6 Eurecat, Centre Tecnològic de Catalunya, Unit of Nutrition and Health, 43204 Reus, Spain

**Keywords:** cafeteria diet, leptin resistance, metabolites, microbiota, obesity, sirtuin

## Abstract

Bioactive compounds such as polyphenols have increased in importance in recent years, and among them, resveratrol (3,5,4′-trihydroxy-trans-stilbene) has generated great interest as an anti-obesity agent. Recent investigations have highlighted the importance of leptin signaling in lipid metabolism in peripheral organs. The aims of this study were (1) to investigate whether resveratrol can reduce fat accumulation in peripheral tissues by increasing their leptin sensitivity and (2) to identify which resveratrol-derived circulating metabolites are potentially involved in these metabolic effects. Serum leptin levels and the leptin signaling pathway were assessed in diet-induced obese rats. Moreover, serum metabolites of resveratrol were studied by ultra-high performance liquid chromatography–mass spectrometry (UHPLC-MS^n^). The daily consumption of 200 mg/kg of resveratrol, but not doses of 50 and 100 mg/kg, reduced body weight and fat accumulation in obese rats and restored leptin sensitivity in the periphery. These effects were due to increases in sirtuin 1 activity in the liver, leptin receptors in muscle and protection against endoplasmic reticulum (ER)-stress in adipose tissue. In general, the resveratrol metabolites associated with these beneficial effects were derived from both phase II and microbiota metabolism, although only those derived from microbiota increased proportionally with the administered dose of resveratrol. In conclusion, resveratrol reversed leptin resistance caused by diet-induced obesity in peripheral organs using tissue-specific mechanisms.

## 1. Introduction

Obesity, defined by the World Health Organization (WHO) as excessive fat accumulation, has been increasing in recent decades and is now reaching epidemic proportions [1]. The increased consumption of energy-dense foods and the significant reduction of physical activity in our daily lives have led to the dysregulation of the homeostatic control of energy balance and, consequently, body weight [2]. The current options for body weight management are energy restriction and physical activity [3]. However, compliance with these treatments is frequently poor, especially in the long term, and thus they are less successful than expected [4]. In this context, the scientific community is interested in naturally occurring bioactive compounds such as polyphenols that may be useful in body weight management [5]. Among these molecules, resveratrol (3,5,4′-trihydroxy-trans-stilbene, RSV), a 14-carbon skeleton stilbene consisting of two aromatic rings with hydroxyl groups in position 3, 5, and 4′, joined by a double styrene bond, has been found to provide a wide range of benefits for many metabolic diseases, including cardiovascular and neurological protective, thermogenic, antioxidant, anti-inflammatory, antiviral and anticancer activities [6,7,8,9,10,11]. Over recent years, these properties have been widely studied in animal and human models, both in vitro and in vivo. Additionally, in most studies in rodent models of diet-induced obesity, RSV alleviated the effects of this dietary pattern [12,13,14]. In addition, RSV is a well-described activator of NAD^+^-dependent deacetylase sirtuin 1 (SIRT1) [15] and adenosine monophosphate-(AMP)-activated protein kinase (AMPK) [16], both of which are considered metabolic sensors that act on gene expression according to the metabolic state of the cell and are closely linked with the benefits of caloric restriction [17].

Leptin, a hormone secreted mainly from white adipose tissue, is the main messenger that carries information about peripheral energy stores to the hypothalamus [18]. The interaction of leptin with its longest receptor isoform (ObRb) promotes the phosphorylation of signal transducer and activator of transcription-3 (STAT3). Subsequently, STAT3 dimerizes and translocates from the cytoplasm into the nucleus, stimulating anorexigenic factors and reducing body weight [19]. For this reason, the role of leptin in controlling energy homeostasis has thus far focused on hypothalamic receptors and neuroendocrine signaling pathways [20,21]. However, accumulating evidence indicates that leptin’s effects on energy balance are also mediated by direct peripheral actions on key metabolic organs such as the liver, skeletal muscle, and adipose tissue [22]. In fact, several studies have recently indicated that peripheral leptin signaling regulates cellular lipid balance to stimulate lipolysis and fatty acid oxidation in white adipose tissues [22,23,24] and skeletal muscle [22,25], decrease triglyceride content and secretion rates in liver [22,26], and even suppress insulin expression and secretion in pancreatic β-cells [22]. However, leptin is unable to exert its effect during diet-induced obesity, and several molecular alterations have been associated with attenuated leptin/STAT3 signaling. These include enhanced endoplasmic reticulum stress (ER-stress) and inflammation, impaired SIRT1 activity and the overexpression of inhibitory factors such as suppressor of cytokine signaling 3 (SOCS3) and protein-tyrosine phosphatase (PTP1B) [19,27].

In this context, we previously showed that a polyphenol-rich extract from grape seeds could improve peripheral and central leptin signaling by increasing SIRT1 functionality and protecting against neuroinflammation [28]. However, to the best of our knowledge, RSV has not been previously studied for its impact on leptin signaling in these organs. Therefore, the aim of the present study was to examine whether RSV exerts part of its anti-obesity effect by modulating leptin sensitivity in the liver, skeletal muscle and adipose tissue. Thus, both serum leptin concentrations and the leptin/STAT3 signaling pathway were evaluated in diet-induced obese animals to investigate the effects of this compound in hyperleptinemic animals with impaired leptin signaling. In addition, as RSV is quickly metabolized by both phase II enzymes and gut microbiota, it was necessary to simultaneously analyze its derived circulating metabolites to obtain a better understanding of the mechanism of action of this compound. 

## 2. Materials and Methods 

### 2.1. Animal Handling

The study was conducted in accordance with the Declaration of Helsinki and was approved by the Ethics Review Committee for Animal Experimentation of the Universitat Rovira i Virgili (reference number 4249 by Generalitat de Catalunya). Male Wistar rats (*n* = 30; 200 ± 50 g body weight) were purchased from Charles River Laboratories (Barcelona, Spain). The animals were housed in pairs under a 12 h light-dark cycle at 22 °C, fed a standard chow diet (Panlab A04, Barcelona, Spain) ad libitum, and were provided access to tap water during the adaptation week. Then, the animals were distributed into equal groups composed of 6 rats. One group was fed a standard chow diet (STD group) with a calorie breakdown of 14% protein, 8% fat and 73% carbohydrates, while the others were fed an STD plus cafeteria diet (CAF group). The CAF diet was composed of 14% protein, 35% fat and 51% carbohydrates and consisted of bacon, carrots, cookies, foie gras, cupcakes, cheese and sugary milk. Nine weeks later, an oral treatment with RSV (Fagron Iberica, Barcelona, Spain) was administered together with the CAF diet for 22 days. The treatment groups were supplemented daily with 50, 100 or 200 mg/kg body weight of RSV dissolved in low-fat sugary milk diluted 1:1 in water. The STD and CAF diet groups were supplemented with the same quantity of vehicle (750 µL) (Figure S1, Supplemental Data). Before supplementation, all rats were trained to voluntarily lick the milk to avoid oral gavage. On the day of sacrifice, the rats received vehicle or RSV and then were fasted for 3 h before sacrifice by decapitation. Blood was collected, and the serum was obtained by centrifugation (1500× *g*, 4 °C and 15 min) and stored at −80 °C. Metabolic tissues such as the liver, calf skeletal muscle and epididymal and retroperitoneal white adipose tissues (eWAT and rWAT, respectively) were excised, weighed, immediately frozen in liquid nitrogen and stored at −80 °C until further analysis.

### 2.2. Body Weight and Composition Analysis

Body weight was monitored weekly until the end of the experiment. In addition, the day before sacrifice, total body composition in live animals was assessed by nuclear magnetic resonance (NMR) using an EchoMRI-700 system (Echo Medical Systems, Houston, TX, USA). Direct measurements of fat mass were obtained in triplicate for each animal, and the results were expressed as a percentage of total body weight.

### 2.3. Hormonal and Metabolic Serum Parameters

Serum glucose (Ref. #998282), total cholesterol (TC) (Ref. #995280) and triacylglycerol (TAG) (Ref. #992320) were measured by enzymatic colorimetric kits (QCA, Barcelona, Spain). Serum leptin (Ref. #EZRL-83K) and insulin (Ref. #EZRMI-13K) concentrations were measured using ELISA kits (Millipore, Madrid, Spain) according to the manufacturer’s instructions.

### 2.4. Tissue Lipid Analysis

The total lipid content in liver, calf skeletal muscle and eWAT was extracted using the Folch method [29]. Briefly, 0.5 g of either liver or eWAT or 0.1 g of calf skeletal muscle was homogenized with 0.45% NaCl in chloroform:methanol (2:1) in an orbital shaker at 4 °C overnight. Then, the homogenate was filtered and washed with 0.45% NaCl solution and 0.9% NaCl solution. An aliquot of each extract was subjected to gravimetric analysis to measure the total lipid concentration. The remainder was allowed to evaporate under nitrogen flow, dissolved in isopropanol and stored at −80 °C until further analysis. The TAG and TC concentrations from the extracts were also measured using QCA enzymatic colorimetric kits (QCA).

### 2.5. Leptin Signaling Analysis

Leptin signaling in the liver, calf skeletal muscle and eWAT was assessed by calculating the activation of STAT3 using an ELISA kit (Abcam, Cambridge, UK)with a phospho-specific antibody for STAT3 phosphorylation (pSTAT3) at tyrosine 705. Briefly, 100 μL of the positive control or sample homogenate was added to wells in duplicate and incubated at room temperature for 2.5 h on an orbital microtiter plate shaker. After washing, 100 μL of the anti-pSTAT3 antibody was applied, and the plate was sealed and incubated for 1 h with shaking. After washing, 100 μL of horseradish peroxidase (HRP)-conjugated anti-rabbit IgG against rabbit anti-pSTAT3 was applied, and the plate was sealed and incubated for 1 h with shaking. Then, the wells were washed, and the 3,3′,5,5′-tetramethylbenzidine (TMB) one-step substrate reagent was incubated for 30 min in the dark. Finally, 50 μL of the stop solution was added, and the plates were immediately read at 450 nm on an microplate automatic plate reader (BioTek, Winooski, VT, USA).

### 2.6. Leptin Sensitivity Index

As cellular pSTAT3 levels are mainly attributable to leptin action in peripheral tissues, leptin sensitivity in the liver, calf skeletal muscle and eWAT was objectively estimated as the ratio of pSTAT3 levels in each tissue to the leptin concentration in serum.

### 2.7. qRT-PCR Analysis

Total RNA was extracted from the liver, calf skeletal muscle and eWAT using TRIzol LS Reagent (Thermo Fisher, Madrid, Spain) and RNeasy Mini Kit (Qiagen, Madrid, Spain) according to the manufacturers’ protocols. The quantity and purity of RNA were measured using a NanoDrop 1000 Spectrophotometer (Thermo Scientific, Madrid, Spain). Only samples with an adequate RNA concentration (A260/A280 ≥ 1.8) and purity (A230/A260 ≥ 2.0) were selected for reverse transcription. Complementary DNA (cDNA) was generated using the High-Capacity cDNA Reverse Transcription Kit (Thermo Fisher), and 10 ng was subjected to quantitative PCR (qPCR) with iTaq Universal SYBR Green Supermix (Bio-Rad, Barcelona, Spain) using the 7900HT Real-Time PCR system (Applied Biosystems, Foster City, CA, USA). The thermal profile settings were 50 °C for 2 min, 95 °C for 2 min, and then 40 cycles at 95 °C for 15 s and 60 °C for 2 min. The forward (FW) and reverse (RV) primers used in this study were obtained from Biomers.net (Ulm, Germany) and can be found in Table S1 in the Supplemental Data. A cycle threshold (Ct) value was generated by setting the threshold during the geometric phase of the cDNA sample amplification. The relative expression of each gene was calculated by referring to cyclophilin peptidylprolyl isomerase A (*Ppia*) mRNA levels and normalized to the STD group. The ΔΔCt method was used and corrected for primer efficiency [30]. Only samples with a quantification cycle lower than 35 were used for fold change calculation.

### 2.8. Western Blot Analysis

Protein levels of the ObRb leptin receptor isoform in the liver, calf skeletal muscle and eWAT were determined by western blot analysis. Tissues were homogenized at 4 °C in 800–1000 µL of radio-immunoprecipitation assay (RIPA) lysis buffer (100 mM Tris-HCl and 300 mM NaCl pH 7.4, 10% Tween, 10% Na-Deox) containing protease and phosphatase inhibitor cocktails using a TissueLyser LT (Qiagen, Madrid, Spain). The homogenate was incubated for 30 min at 4 °C and then centrifuged at 12,000× *g* for 20 min at 4 °C. The supernatant was placed in fresh tubes and used to determine total protein and for immunoblotting analyses. The total protein content of the supernatant was measured using the Pierce bicinchoninic acid assay (BCA) protein assay kit (Thermo Scientific, Madrid, Spain). Samples were denatured by mixing with loading buffer solution (Tris-HCl 0.5 M pH 6.8, glycerol, sodium dodecyl sulfate (SDS), β-mercaptoethanol and Bromophenol Blue) and then heated at 99 °C for 5 min in a thermocycler (Multigen Labnet, Barcelona, Spain). Acrylamide gels were prepared using TGX Fast Cast Acrylamide Kit(Bio-Rad, Barcelona, Spain), and 25 µg of protein was subjected to SDS-polyacrylamide gel electrophoresis (PAGE) in electrophoresis buffer (glycine 192 mM, Tris base 25 mM and 1% SDS). Proteins were electrotransferred onto supported polyvinylidene difluoride (PVDF) membranes (Trans-Blot Turbo Mini PVDF Transfer Packs, Bio-Rad). After blocking with 5% non-fat dried milk, the membranes were incubated with gentle agitation overnight at 4 °C with a specific antibody for ObRb (ab177469, Abcam, Cambridge, UK) diluted 1:1000. For β-actin analysis as a loading control, membranes were incubated with a rabbit anti-actin primary antibody (A2066, Sigma-Aldrich, Madrid, Spain) diluted 1:1000. Finally, membranes were incubated with anti-rabbit horseradish peroxidase secondary antibody (NA9344, GE Healthcare, Barcelona, Spain) diluted 1:10,000. Protein levels were detected with the chemiluminescent detection reagent ECL Select (GE Healthcare, Barcelona, Spain) and GeneSys image acquisition software (G:Box series, Syngene, Barcelona, Spain). The protein bands were quantitated by densitometry using ImageJ software (W.S Rasband, Bethesda, MD, USA), and each band was normalized by the corresponding β-actin band, and finally, the treatment groups were normalized by the STD group.

### 2.9. SIRT1 Activity Assay

The SIRT1 activity in liver, calf skeletal muscles and eWAT was determined using a SIRT1 direct fluorescent screening assay kit (Cayman, Ann Arbor, MI, USA) as previously described [31]. Briefly, a total of 25 μL of assay buffer (50 mM Tris-HCl, pH 8.0, containing 137 mM NaCl, 2.7 mM KCl, and 1 mM MgCl_2_), 5 μL of tissue extract (1.5 mg/mL), and 15 μL of substrate (Arg-His-Lys-Lys(ε-acetyl)--7-amino-4-methylcoumarin) solution were added to all wells. The fluorescence intensity was monitored every 2 min for 1 h using a BertholdTech TriStar2S fluorescence plate reader (Berthold Technologies, Bad Wildbad, Germany) at an excitation wavelength of 355 nm and an emission wavelength of 460 nm. The results were expressed as the rate of reaction for the first 30 min, when there was a linear relationship between fluorescence and time.

### 2.10. Resveratrol Metabolite Extraction from Serum Samples

Serum samples were extracted as previously reported by Savi et al. [32] with minor modifications. Briefly, 300 μL of serum was diluted with 1 mL of acidified acetonitrile (2% formic acid, Sigma-Aldrich, Madrid, Spain). The samples were vortexed vigorously, ultrasonicated for 10 min, and centrifuged at 12,000 rpm for 5 min. Then, the supernatant was dried under vacuum by rotary evaporation, and the pellet was suspended in 100 μL of methanol 50% (*v*/*v*) acidified with formic acid 0.1% (*v*/*v*) and centrifuged at 12,000 rpm for 5 min prior to analysis by ultra-high performance liquid chromatography–mass spectrometry (UHPLC-MS). 

### 2.11. UHPLC-MS^n^ Analysis

Samples were analyzed by an Accela UHPLC 1250 coupled to a linear ion trap-mass spectrometer (LTQ XL, Thermo Fisher Scientific Inc., San Jose, CA, USA) fitted with a heated-electrospray ionization source (H-ESI-II; Thermo Fisher Scientific Inc., Madrid, Spain). The chromatographic and ionization parameters for the analysis of the samples were set as previously described [33]. Metabolite identification was performed by comparing the retention time with authentic standards and/or MS^n^ fragmentation patterns in negative ionization mode (Table S2 in Supplemental Data). The glucuronide forms of RSV and dihydroresveratrol (DRSV) were fragmented using a collision-induced dissociation (CID) value of 16 (arbitrary units), whereas aglycones and sulfate conjugates required CID values of 34 and 23, respectively. Pure helium gas was used for CID. Data processing was performed using Xcalibur software from Thermo Scientific (Madrid, Spain). Quantification was performed using specific MS2 full scans and calibration curves of pure standards in the case of RSV, resveratrol-3-sulfate (R3S), resveratrol-4′-sulfate (R4S), resveratrol-3-glucuronide (R3G) and DRSV. Calibration curves were prepared, in the range of expected concentrations, by supplementation with known concentrations of available standards. When a standard was not available, the conjugated metabolites were quantified based on the most structurally similar compound and expressed as their equivalents. All of them were quantified using a nine-point calibration curve which ranged from 0.1 to 100 µM. Spiked samples were extracted and subsequently analysed by using the same procedure as the serum samples. The calibration curves were finally generated for each standard by plotting the peak abundance versus the concentration and fitting to a linear regression. Quality parameters were determined to validate and evaluate the suitability of the developed quantitative method as was done previously [33]. 

### 2.12. Statistical Analysis

The data are expressed as the means ± standard errors of the means (SEM). Groups were compared by Student’s *t*-test or two-way ANOVA and Bonferroni’s test. Outliers were determined by Grubbs’ test. MetaboAnalyst (Xia Lab, McGill University, Montréal, Quebec, Canada) was used to perform multivariate statistical analyses. Correlation analysis was performed using the nonparametric Spearman test. Statistical analyses were performed using XLSTAT 2017 (Addinsoft, Paris, France). Graphics were prepared using GraphPad Prism 6 (GraphPad Software, San Diego, CA, USA). *p* < 0.05 was considered statistically significant, and *p* < 0.1 was considered to indicate a tendency. 

## 3. Results

### 3.1. RSV Attenuates Diet-Induced Body Fat Increase, Hypertriglyceridemia and Hyperleptinemia

**Figure 1:**
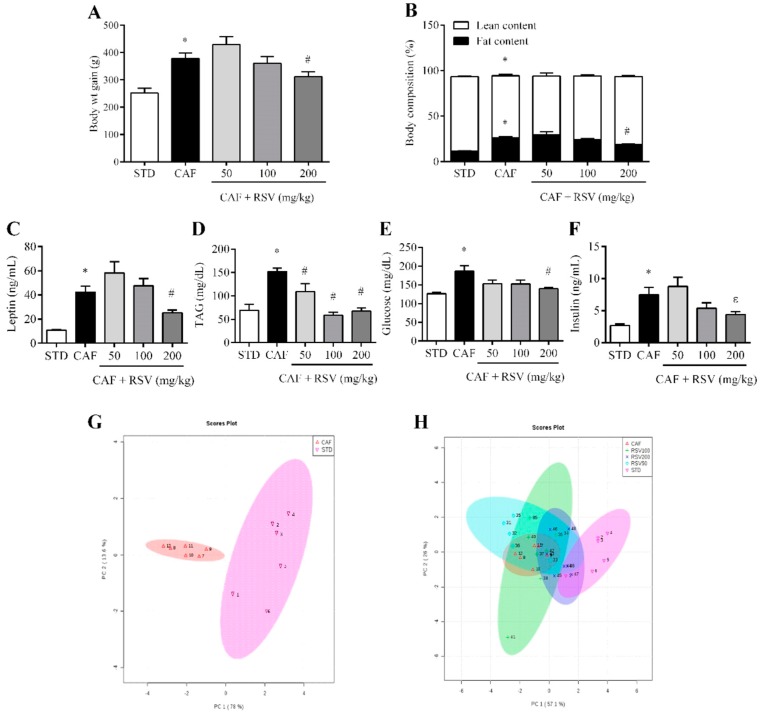
Metabolic parameters. The rats were fed the STD or CAF diet for 9 weeks. Then, the rats in the STD and CAF groups were treated orally with RSV (50, 100 or 200 mg per kg of body weight) or vehicle for 3 weeks. (**A**) Body weight gain (g) from the first day of the experiment until the last day; (**B**) Body composition (%) assessed by NMR, including fat and lean content; Serological levels of (**C**) leptin, (**D**) TAG, (**E**) glucose and (**F**) insulin. (**G**) and (**H**) are PCAs representing the clusters between the different groups according to the studied biometric parameters. Data are expressed as the mean ± SEM, *n* = 6. * denotes *p* < 0.05, Student’s *t*-test comparing the CAF group to the STD group. ^#^ denotes *p* < 0.05 and ^ε^ *p* < 0.1, Student’s *t*-test comparing the RSV group to the CAF group. CAF: cafeteria diet; NMR: nuclear magnetic resonance; RSV: resveratrol; STD: standard chow diet; TAG: triacylglycerol.

The CAF diet for 12 weeks consistently resulted in obesity, as indicated by the significantly higher body weight gain (50.1% higher) and total body fat mass (124.3% higher, assessed by NMR scanning) compared to the STD group. Notably, the body weight gain was 17% lower in animals supplemented with RSV at 200 mg/kg daily compared to the CAF group (Figure 1A), and this reduction was associated with a significant decrease in total body fat mass (Figure 1B). Notably, at this dose, no differences were found among groups in food intake (data not shown). Importantly, the consumption of 200 mg/kg of RSV partially reversed the hyperleptinemia induced by CAF diet (40.1% lower) (Figure 1C), reinforcing the robust metabolic correlation between leptin levels and total body fat mass in our experimental model (ρ = 0.93, *p* < 0.05). In addition, at this dose, RSV was also effective in normalizing serum concentrations of TAG, glucose and insulin in a fasting state (Figure 1D–F), indicating that RSV has an insulin-sensitizing effect in diet-induced obesity. By contrast, the daily consumption of 50 and 100 mg/kg of RSV for 22 days did not exert any beneficial effects with respect to body weight, total body fat accumulation and hormonal and metabolic serum parameters. 

### 3.2. Multivariate Analysis Shows That RSV Partially Reverses the Metabolic Alterations Induced by the Cafeteria Diet

To further evaluate the effect of RSV from a multivariate point of view, principal component analysis (PCA) was performed to analyze globally the distribution of animals among all anthropometric, metabolic and biochemical variables. Accordingly, the PCA score plot for the STD and CAF groups accounted for 91.6% of the variance of the original matrix, and each animal was clearly clustered according to their diet (Figure 1G). In addition, when the multivariate analysis was used to evaluate the effect of RSV consumption on diet-induced obesity, we observed that animals treated at doses of 50 and 100 mg/kg could not be clustered separately with respect to CAF animals, and only animals daily supplemented at a dose of 200 mg/kg were clustered in an intermediate position between the CAF and STD groups, indicating that RSV at this dose could exert a tendency to reverse the metabolic alterations induced by an obesogenic diet and a return to the basal situation (Figure 1H).

### 3.3. RSV Decreases Diet-Induced Lipid Content in Liver, Skeletal Muscle and Adipose Tissues

**Figure 2:**
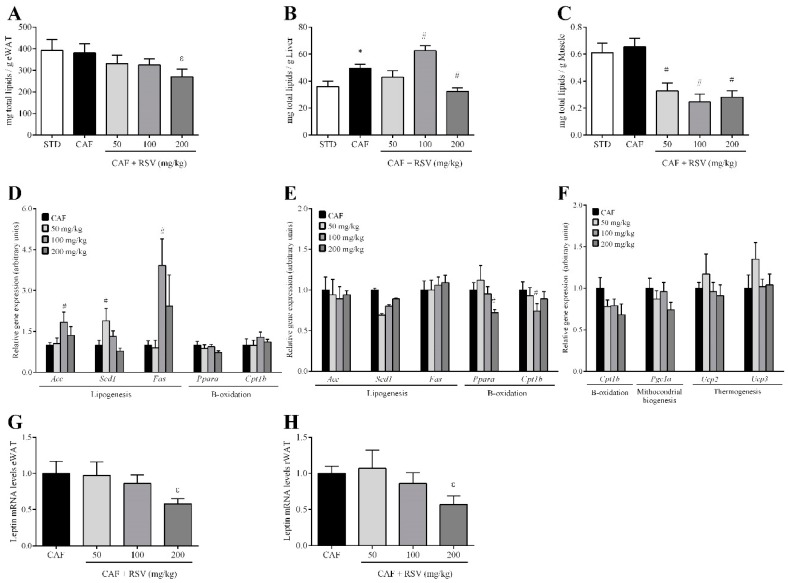
Lipid profile. The rats were fed the STD or CAF diet for 9 weeks. Then, the rats in the STD and CAF groups were treated orally with RSV (50, 100 or 200 mg per kg of body weight) for 3 weeks. Total lipids in (**A**) eWAT, (**B**) liver and (**C**) calf skeletal muscle in mg for each g of tissue. (**D**) Expression in the liver of genes related to lipogenesis (*Acc*, *Scd1* and *Fas*) and β-oxidation (*Ppara* and *Cpt1b*). (**E**) Expression in eWAT of genes related to lipogenesis (*Acc*, *Scd1* and *Fas*) and β-oxidation (*Ppara* and *Cpt1b*). (**F**) Expression in calf skeletal muscle of genes related to β-oxidation (*Cpt1b*), mitochondrial biogenesis (*Pgc1a*) and thermogenesis (*Ucp2* and *Ucp3*). Leptin gene expression in (**G**) eWAT and (**H**) rWAT. Data are expressed as the mean ± SEM, *n* = 6. * denotes *p* < 0.05, Student’s *t*-test comparing the CAF group to the STD group. ^#^ indicates *p* < 0.05 and ^ε^ *p* < 0.1, Student’s *t*-test comparing the RSV group to the CAF group. CAF: cafeteria diet; eWAT: epididymal white adipose tissue; RSV: resveratrol; rWAT: retroperitoneal white adipose tissue; STD: standard chow diet. *Acc* (acetyl-CoA carboxylase); *Cpt1b* (carnitine palmitoyltransferase 1b); *Fas* (fatty acid synthase); *Pgc1a* (peroxisome proliferator-activated receptor gamma coactivator 1-alpha), *Ppara* (peroxisome proliferator activated receptor alpha); *Scd1* (stearoyl-CoA desaturase 1); *Ucp2* (mitochondrial uncoupling protein 2); *Ucp3* (mitochondrial uncoupling protein 3).

To assess the contribution of visceral fat accumulation to the decrease in total body fat mass, we next evaluated the effect of RSV on fat deposition in three important metabolic peripheral tissues: visceral white adipose tissue, liver and skeletal muscle. Again, the CAF diet for 12 weeks resulted in a significant increase in two different visceral WAT depots compared to the STD group, including eWAT (18.6 ± 1.4 vs. 8.9 ± 0.9 g, respectively) and retroperitoneal WAT (rWAT, 8.4 ± 0.9 vs. 3.6 ± 0.1 g, respectively). Notably, at a dose of 200 mg/kg, RSV elicited a significant decrease in the weights of these depots compared with CAF animals, and this effect was more evident in eWAT (14.6 ± 1.4 g, 21% lower) than in rWAT (7.1 ± 0.7 g, 16% lower). In addition, RSV also tended to reduce the total fat content in eWAT (Figure 2A) and significantly in the liver (Figure 2B), but above all in the skeletal muscle, although not in a dose-dependent manner (Figure 2C). Interestingly, this decrease in fat depots in peripheral organs was directly associated with significant reductions of both cholesterol and TAG content (Table S3, Supplemental Data), and in turn, it was positively and significantly related to serum leptin levels in the liver (ρ = 0.54, *p* < 0.05) and eWAT (ρ = 0.43, *p* < 0.05 in eWAT), implicating leptin in the regulation of lipid metabolism in peripheral tissues.

### 3.4. RSV Directly Down-Regulates Leptin Transcription in Adipose Tissues

To further examine the mechanism by which RSV regulates lipid accumulation in peripheral tissues, we assessed the gene expression of lipid-regulating enzymes by RT-qPCR in the liver, skeletal muscle and eWAT. Completely contrary to our expectations, we found that the expression levels of genes involved in lipogenesis but not fatty acid oxidation, such as *Acc*, *Scd1* and *Fas*, were significantly increased in the liver of animals supplemented with 50 and 100 mg/kg of RSV (Figure 2D). By contrast, no significant changes were observed in the expression of genes encoding enzymes for lipogenesis, whereas RSV down-regulated fatty acid oxidation in eWAT (Figure 2E). Moreover, we did not observe any significant changes in thermogenesis, mitochondrial biogenesis and fatty acid oxidation in skeletal muscle (Figure 2F). Importantly, in eWAT and rWAT, at a dose of 200 mg/kg, RSV consumption tended to down-regulate leptin mRNA levels compared with CAF animals (Figure 2G,H), indicating that animals undergoing RSV treatment more efficiently regulated leptin production and secretion in these tissues than those in the CAF group.

### 3.5. RSV Potentiates Leptin Sensitivity in Liver, Skeletal Muscle and Adipose Tissue

**Figure 3:**
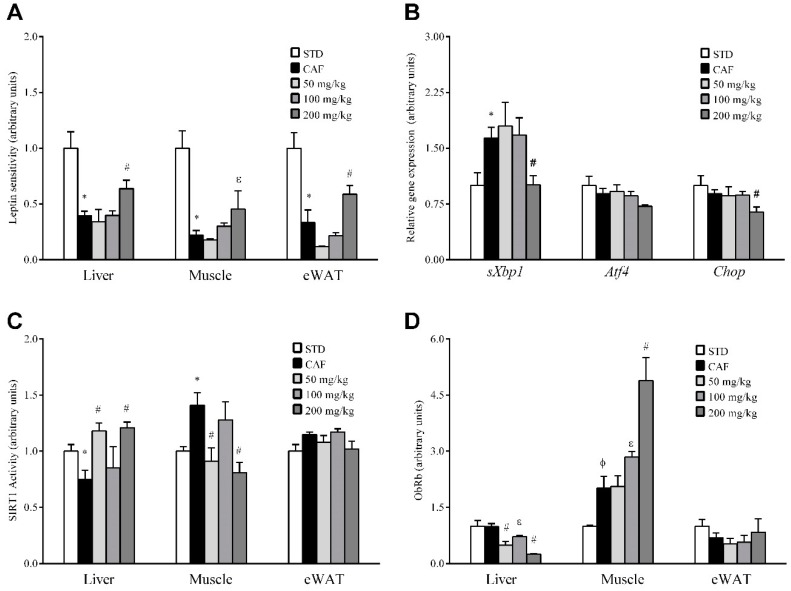
Leptin sensitivity and signaling. The rats were fed the STD or CAF diet for 9 weeks. Then, the STD and CAF rats were treated orally with RSV (50, 100 or 200 mg per kg of body wt) or vehicle for 3 weeks. (**A**) The leptin sensitivity index. (**B**) Gene expression of ER-stress markers in eWAT. (**C**) SIRT1 activity. (**D**) WB results for ObRb. Data are expressed as the mean ± SEM, *n* = 6. * *p* < 0.05 and ^φ^ *p* < 0.1, Student’s *t*-test comparing the CAF group with the STD group. ^#^ *p* < 0.05 and ^ε^ *p* < 0.1, Student’s *t*-test comparing the RSV group with the CAF group. CAF: cafeteria diet; ER: endoplasmic reticulum; eWAT: epididymal white adipose tissue; LSI: leptin sensitivity index; ObRb: leptin receptor isoform b; RSV: resveratrol; SIRT1: NAD^+^-dependent deacetylase sirtuin-1; STD: standard chow diet; VH: vehicle; WB: western blot; wt: weight. *Atf4* (activating transcription factor 4), *Chop* (DNA damage inducible transcript 3), s*Xbp1* (spliced x-box binding protein 1).

To determine if the observed decreases in leptin production and circulating levels could indicate that RSV directly affects leptin signaling in peripheral tissues, we assessed leptin sensitivity in liver, skeletal muscle and eWAT by detecting STAT3 activation (pSTAT3). Because pSTAT3 levels are mainly attributable to leptin action in theses tissues, we assessed the ratio of tissue-specific levels of pSTAT3 to the circulating leptin concentration to estimate the degree of sensitivity of each tissue to this hormone. In this context, the leptin sensitivity of CAF animals was significantly reduced compared to the STD group in all three tissues studied, and importantly, when RSV was administered at dose of 200 mg/kg, the leptin sensitivity significantly increased to basal levels, indicating partial reversion of the situation observed in CAF animals (Figure 3A). By contrast, no significant effects on leptin sensitivity were observed at lower doses in any of the tissues assessed. 

Next, we studied the gene expression levels of Socs3 and Ptp1b, negative feedback regulatory molecules involved in leptin signaling, by qRT-PCR. However, *Socs3* and *Ptp1b* mRNA levels were not significantly altered by RSV consumption (Figure S2, Supplemental Data). Finally, we also investigated the impact of RSV on two metabolic processes closely associated with leptin signaling disruption: local inflammation and ER stress. However, *iNos* mRNA expression levels were not significantly regulated in any tissue (Figure S3, Supplemental Data). In a similar manner, in liver and muscle, transcripts related to ER stress were not modulated in any group of animals undergoing RSV supplementation (Figure S4, Supplemental Data). Interestingly, a significant decrease in ER-stress markers was observed in eWAT in animals under the highest dose of RSV (Figure 3B).

### 3.6. RSV Distinctively Modulates Sirtuin-1 (SIRT1) Activity and Leptin Receptor (ObRb) Protein Expression in Peripheral Tissues

To elucidate the molecular mechanisms by which RSV potentially rescues leptin sensitivity in these tissues, we next evaluated whether RSV consumption could result in enhanced SIRT1 functionality, which could be an additional mechanism involved in the regulation of leptin signal transduction in the periphery. Thus, we analyzed the deacetylase activity of SIRT1 in liver, skeletal muscle and eWAT (Figure 3C). Notably, robust activation of SIRT1 was observed in the liver of animals supplemented with 50 and 200 mg/kg of RSV, indicating that a relatively low dose of RSV (50 mg/kg) is sufficient to efficiently activate this enzyme in the liver. By contrast, at these same doses, SIRT1 activity was notably decreased in skeletal muscle and was not significantly affected in eWAT, suggesting that if RSV is a true leptin sensitizer, this activity is not mediated by an increase in SIRT1 functionality in skeletal muscle and eWAT. Therefore, we also investigated by immunoblotting whether the modulation of leptin sensitivity in these tissues could also be directly mediated by increasing the cell content of the long leptin receptor isoform ObRb (Figure 3D). Interestingly, the consumption of RSV resulted in a dose-dependent significant increase in ObRb protein levels in skeletal muscle, although statistically significant differences were only observed at a dose of 200 mg/kg. Importantly, in contrast to skeletal muscle, ObRb protein levels in liver were decreased significantly in animals under RSV at doses of 50 and 200 mg/kg, whereas in eWAT, ObRb protein levels were not significantly affected at any dose. These results indicate that RSV can modulate different cellular processes in a tissue-specific manner.

### 3.7. Different RSV Metabolites, Including Microbial and Phase II Conjugates, Could Explain the Body Fat-Lowering Effects of RSV Consumption

**Figure 4:**
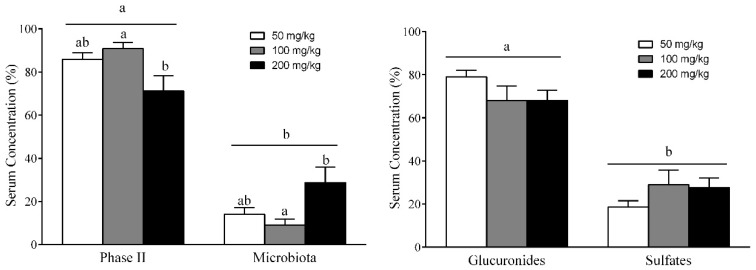
Serum resveratrol metabolites. The rats were fed the STD or CAF diet for 9 weeks. Then, the STD and CAF rats were treated orally with RSV (50, 100 or 200 mg per kg of body wt) or vehicle for 3 weeks. The metabolites present in serum were classified as (**A**) phase II RSV metabolites or microbial metabolites and as (**B**) glucuronide or sulfate metabolites. Data are expressed as the mean ± SEM, *n* = 6. ^a,b^ denote significant differences between groups (*p* < 0.05; two-way ANOVA and Bonferroni’s test).

Since the efficacy of orally administered RSV depends on its absorption and metabolism, we next investigated whether RSV and its metabolites found in the bloodstream can account for the observed anti-obesity effects after the daily consumption of RSV for 22 days. Table 1 details the serum concentrations of each metabolite of RSV. The administration of RSV at 50, 100 and 200 mg/kg led to high serum concentrations of some metabolites, in the range of µM. Interestingly, 10 different RSV-derived metabolites, but not the parent compound, were detected in serum 3 h after the last RSV treatment. These metabolites included seven phase II metabolites of RSV (R3G, R4G, RDG, R3S, R4S, RDS and RSG) and three gut microbiota-derived metabolites, including the glucuronide and sulfate conjugates of DRSV (DRG, DRS and DRGS). When the serum distribution of these two types of metabolites was analyzed, phase II RSV metabolites were found to be predominant over DRSV metabolites derived from microbiota by more than two fold (Figure 4A). Interestingly, the concentration of microbial DRSV metabolites significantly increased at a dose of 200 mg/kg, whereas the opposite occurred for phase II RSV metabolites. Thus, the largest circulating levels of microbial metabolites were found at the highest dose of 200 mg/kg. In addition, total glucuronide metabolites (R3G, R4G, RDG and DRG) were also detected in higher levels than total sulfate conjugates (R3S, R4S, RDS and DRS) at all doses (Figure 4B). However, the concentration of glucuronide conjugates tended to decrease when the RSV dosage was increased, whereas sulfate metabolites significantly increased at doses of 100 and 200 mg/kg. 

Finally, to determine which blood RSV metabolites could potentially be involved in the anti-obesity effects of RSV, we used the Spearman’s correlation test to evaluate the relationship of RSV metabolites with body and fat mass as well as with leptin sensitivity in each peripheral tissue (Table S4, Supplemental Data). R4G and R3S were the only phase II RSV metabolites that showed significant and negative correlations with total body fat mass (ρ = −0.67 and −0.76, *p* = 0.033 and 0.011, respectively) and circulating leptin levels (ρ = −0.66 and −0.60, *p* = 0.038 and 0.067, respectively). In addition, R4G was also positively associated with leptin sensitivity in liver (ρ = 0.72, *p* = 0.03), skeletal muscle (ρ = 0.69, *p* = 0.05) and adipose tissue (ρ = 0.79, *p* = 0.021), whereas R4S was positively associated with leptin sensitivity in skeletal muscle (ρ = 0.81, *p* = 0.015) and adipose tissue (ρ = 0.79, *p* = 0.021). When the correlation coefficients were analyzed for the microbial DRSV metabolites, only DRSG presented a negative and significant correlation with diet-induced body weight increase (ρ = 0.66, *p* = 0.038). In addition, DRSG was related to leptin sensitivity in skeletal muscle (ρ = 0.81, *p* = 0.015) and adipose tissue (ρ = 0.71, *p* = 0.047), whereas DRS was related to leptin sensitivity in skeletal muscle (ρ = 0.69, *p* = 0.05).

## 4. Discussion

Previous studies by our group indicated that chronic consumption of grape-seed proanthocyanidins for three weeks by diet-induced obese rats significantly decreased both hepatic fat content and circulating plasmatic leptin levels, presumably by restoring SIRT1 functionality and leptin signaling in both the hypothalamus and liver [28,31]. Nonetheless, studies of other compounds with complementary or more powerful effects are necessary to combat metabolic diseases associated with leptin dysfunction, such as obesity. Accordingly, in the present study, we demonstrated that RSV, a dietary non-flavonoid polyphenol found in grapes and red wine, decreased body fat mass and leptinemia by restoring leptin sensitivity in the liver, skeletal muscle and adipose tissue. 

Leptin is a pleiotropic hormone with a variety of functions within the organism and activity in different tissues. Liver and skeletal muscle are the tissues with greatest metabolic activity and, together with adipose tissue, constitute important targets for the leptin regulation of lipid metabolism [34,35]. However, pathological states such as obesity have been related to peripheral leptin resistance development, and dietary components have been proposed to modulate leptin actions in these peripheral tissues, suggesting that leptin resistance may also result from specific nutrient intake [36,37]. In this sense, our CAF-induced obesity rat model exhibited body weight/fat increase, hyperleptinemia and peripheral leptin resistance as indicated by the impairment of leptin-induced STAT3 phosphorylation in these tissues. pSTAT3 levels are widely studied to evaluate leptin sensitivity as STAT3 is proportionally activated by leptin concentrations in these tissues [38].

Remarkably, our results showed an ability of RSV at 200 mg/kg to normalize tissue fat content and leptin expression and secretion as well as to enhance peripheral leptin sensitivity, highlighting the overall beneficial effect of RSV in the modulation of diet-induced obesity at this dose. Conversely, RSV did not regulate the gene expression of enzymes directly involved in peripheral lipid metabolism, such as *Acc*, *Fas*, *Cpt1b* and *Scd1*. Nevertheless, other possibilities cannot be discarded, such as that RSV could regulate the proteins involved in fatty acid oxidation by post-transcriptional mechanisms that are not detectable by qRT-PCR analysis, or that RSV could induce lipid oxidation in tissues other than those examined in this study. Alternatively, RSV could also induce the catabolism of fatty acids to ketone bodies as previously suggested in Csb^m/m^ mice fed a standard diet supplemented with 100 mg/kg_chow_ RSV ad libitum [39]. In addition, RSV did not down-regulate the gene expression of relevant enzymes involved in leptin signaling, such as Socs3 and Ptp1b. Contradictory results have been published about the effect of RSV on these markers of leptin signaling in different tissues [40,41,42], indicating that the duration of the treatment and the grade of obesity achieved can directly influence the effect of RSV in these tissues.

The daily consumption of 50 and 100 mg/kg of RSV for 22 days in combination with the CAF diet did not change any metabolic parameter or leptin sensitivity in our experimental model. Some contradictory results have been published about the effectiveness of RSV on metabolic alterations in rodents. Andrade et al. reported that the consumption of 30 mg/kg of RSV by FVN/N mice for 60 days in combination with a CAF-rich diet exerted beneficial effects on body fat and weight [43]. By contrast, supplementation of diet-induced obese C57BL/6J mice with 22.5 and 45 mg/kg of RSV for 12 weeks [44] or 200 mg/kg for 20 weeks [45] did not cause any significant change in body weight, indicating that the effect of RSV in rodents might depend on the treatment length, RSV dosage and the percentage of fat present in the diet. In our study, the impact of the CAF diet was too robust, as the lowest doses of RSV administered could not counteract the diet-induced dysregulation of lipid metabolism and leptin signaling.

Other mechanisms have been proposed to explain the effects of phenolic compounds on body weight in mammals, including their inhibitory effects on food intake and fat absorption as well as on intestinal permeability and gut microbiota. However, as we did not evaluate the effect of RSV on all of these mechanisms, we cannot be completely sure that the consumption of this compound exclusively prevents fat accumulation through the leptin signaling activation in peripheral tissues. Thus, more studies are needed that elucidate the molecular mechanisms involved in this beneficial effect.

The induction of peripheral leptin resistance in diet-induced models has been primarily attributed to the induction of pro-inflammatory signaling and ER stress [27]. However, in this study, we did not find significant differences in inflammatory status in any of the three tissues studied as indicated by the *iNos* gene expression levels, but important anti-inflammatory effects of RSV in these animals cannot be excluded. In this sense, in contrast to our results, Kimbrough et al. observed the down-regulation of *iNos* by RSV in hepatocytes in an inflammatory experimental model [46], as did Centeno-Baez et al. in muscle and WAT in lipopolysaccharide (LPS)-treated C57BL6 mice [47]. Conversely, our results showed a significant reduction of *sXBP1* gene expression in adipose tissue, suggesting that this local decrease in ER stress in adipocytes is one of the mechanisms by which RSV re-establishes appropriate leptin sensitivity in this tissue. In addition, SIRT1 functionality and ObRb levels have been highlighted as mediators of leptin action in peripheral organs. Thus, both the overexpression of *Sirt1* in the liver and the enhanced *ObRb* protein content in skeletal muscle induced by RSV could be mechanisms by which this compound increases leptin signaling in these tissues. In fact, this beneficial effect of RSV on SIRT1 activity in the liver is in accordance with a previous report [48]. However, the different responses of the liver, muscle and adipose to RSV suggest different functions of SIRT1 and ObRb in peripheral tissues, and thus further studies are required to clarify the molecular mechanism by which RSV regulates leptin signaling in each tissue under obesogenic conditions.

The efficacy of orally administered RSV depends on its absorption and metabolism. RSV is quickly absorbed in the intestine via simple intestinal transepithelial transport and by ATP-dependent binding cassette transporters, but most RSV undergoes rapid and extensive phase II metabolism in enterocytes before entering the blood and further into the liver [49,50]. Accordingly, RSV is mainly converted into glucuronide and sulfate metabolites. Interestingly, in the present study, we detected RSV metabolites but not RSV. Our results are in line with some previously published findings of RSV metabolites but not free RSV in different peripheral tissues when rats were supplemented with 300 [51] or 60 mg/kg [52]. In addition, total glucuronide RSV metabolites (R3G, R4G and RDG) were also detected in higher levels than total sulfate RSV conjugates (R3S, R4S and RDS) at all doses. Similarly, in a previous study using male Sprague–Dawley rats, Marier et al. observed 46 times more glucuronidated forms in plasma than other metabolites 4 h after oral administration of 50 mg/kg of RSV [53]. However, other researchers have reported that the sulfate forms were prevalent over glucuronides in male Wistar rats orally supplemented with 300 mg/kg of RSV for 8 weeks, whereas no RSV conjugates were detected in the group with a dose of 50 mg/kg [51].

Notably, in our study, the concentration of glucuronide RSV conjugates tended to decrease as the RSV dosage increased, whereas the sulfate RSV metabolites increased at the highest doses. Similarly, Andres-Lacueva et al. observed that as the dose of RSV in rats increased (6, 30 and 60 mg/kg/day for 6 weeks), there was an increase in the sulfate forms compared with the glucuronides [52]. These results may suggest that glucuronidation but not sulfation could be a saturable metabolic pathway, at least in the range of doses used in the present study. Nevertheless, the potential degradation of glucuronide metabolites cannot be discarded. Consequently, further studies are needed to better elucidate this issue.

Only a few studies have considered the determination of RSV-derived microbial metabolites after RSV consumption. Consequently, we also assessed DRSV concentrations in serum in free form or as glucuronide and sulfate conjugates. Notably, in our study, the largest circulating levels of microbial metabolites were found at the highest dose of 200 mg/kg. These data could provide a clue to explain the protective effects on body fat accumulation and leptin sensitivity observed only at this dose of RSV. In fact, our results showed negative correlations of levels of DRSG metabolites from microbiota with body fat mass, circulating leptin levels and body weight gain. However, the levels of most of these DRSV metabolites detected in serum were low in comparison to RSV metabolites, and thus it is difficult to understand how they contributed to the effects observed. In addition, some studies also showed that, in 3T3-L1 cells, R3S, R3G and R4G decreased both mRNA and leptin secretion [54], increased the expression of Atgl, Cpt1, Sirt1 and Pgc1a, and decreased the expression of Fas [55]. Consequently, more studies are needed to explain the in vivo effects induced by this polyphenol after long-term treatment.

## 5. Conclusions

In summary, we can conclude that RSV can reverse the disruption of metabolic parameters and the lipid profile in a diet-induced obese rat model. This beneficial effect could be explained by the restoration of leptin sensitivity in the three peripheral organs described as more metabolically active. In the liver, RSV could act via a SIRT1-dependent manner, whereas in muscle and adipose tissue, its action was mediated by increasing ObRb content and protecting against ER stress, respectively. However, further studies are required to clarify the molecular mechanisms by which RSV regulates leptin signaling in obesity. Finally, the metabolites derived from the gut microbiota may partially explain the contribution of the highest dose of RSV to reducing the metabolic alteration caused by obesity.

## Figures and Tables

**Table 1:** The results obtained for each metabolite (µM) present in serum respect each group of rats treated with RSV.

RSV Metabolites (µM)		50 mg/Kg	100 mg/Kg	200 mg/Kg
Phase II	R4G	18.52 ± 4.54	15.45 ± 1.69	20.53 ± 7.73
	R3G	12.44 ± 4.85	8.43 ± 3.79	1.84 ± 0.61 *
	RDG	0.19 ± 0.04	0.29 ± 0.09	0.23 ± 0.04
	R4S	0.12 ± 0.02	0.16 ± 0.05	0.27 ± 0.09
	R3S	7.06 ± 2.66	5.69 ± 1.21	5.59 ± 2.04
	RDS	0.80 ± 0.31	1.39 ± 0.31	0.66 ± 0.09
	RSG	0.83 ± 0.15	1.00 ± 0.14	0.83 ± 0.21
Microbiota	DRG	3.95 ± 0.60	2.72 ± 0.66	9.43 ± 2.28 *
	DRS	1.15 ± 0.46	1.24 ± 0.63	3.92 ± 1.30
	DRSG	0.11 ± 0.02	0.15 ± 0.04	0.21 ± 0.06

Abbreviations: R4G: resveratrol-4′-glucuronide; R3G: resveratrol-3-glucuronide; R3S: resveratrol-3-sulfate; R4S: resveratrol-4′-sulfate; RDS: resveratrol-disulfate; RDG: resveratrol-diglucoronide; RSG: resveratrol-sulfate-glucuronide; DRG: dihydroresveratrol-glucuronide; DRS: dihydroresveratrol-sulfate; DRSG: dihydroresveratrol-sulfate-glucuronide. * *p* < 0.05.

## Supplementary Materials

The following are available online at http://www.mdpi.com/2072-6643/10/11/1757/s1, Table S1: A summary of the rat-specific primer sequences used for qRT-PCR analysis, Table S2: Chromatographic and fragmentation characteristics of RSV metabolites identified by UHPLC-MSn in serum samples, Table S3: Different biochemical parameters of liver, calf skeletal muscle and eWAT, Table S4. Correlation analysis of the most relevant biochemical parameters and the metabolites concentrations of RSV present in the serum of rats treated with 50, 100 or 200 mg/kg RSV, Figure S1: A scheme of the distribution of animals in the study, Figure S2: Inhibitors of the leptin signaling, Figure S3: iNos gene expression, Figure S4: Gene expression analysis of ER-stress markers.
