# Supplementary material for: Potential Involvement of Peripheral Leptin/STAT3 Signaling in the Effects of Resveratrol and Its Metabolites on Reducing Body Fat Accumulation
Source: Nutrients. 2018 Nov 14;10(11):1757. doi: 10.3390/nu10111757 (PMC6265754; doi:10.3390/nu10111757)
Supplement: Supplementary file 1 [file nutrients-10-01757-s001.pdf]

Supplemental data for:

Potential Involvement of Peripheral Leptin/STAT3 Signaling in the Effects of Resveratrol and its Metabolites on Reducing Body Fat Accumulation

Andrea Ardid-Ruiz[1], Maria Ibars[1], Pedro Mena[2], Daniele Del Rio[3,4,5], Begoña Muguerza[1], Cinta Bladé[1], Lluís Arola[1,6], Gerard Aragonès,[1] Manuel Suárez[1]*

[1]Universitat Rovira i Virgili, Department of Biochemistry and Biotechnology, Nutrigenomics Research Group, Tarragona, Spain; [2]Human Nutrition Unit, Department of Food and Drugs, University of Parma, Parma, Italy; [3] Department of Veterinary Medicine, University of Parma, Parma, Italy; [4]School for Advanced Studies on Food and Nutrition, University of Parma, Italy; [5] Microbiome Research Hub, University of Parma; [6]Technological Unit of Nutrition and Health, EURECAT-Technological Center of Catalonia, Reus, Spain.

*Corresponding author: Gerard Aragonès, Universitat Rovira i Virgili, Department of Biochemistry and Biotechnology, Marcel·lí Domingo 1, 43007, Tarragona, Spain. Tel.: +34-977-558-188; Fax: 34-977-558-232; E-mail: gerard.aragones@urv.cat

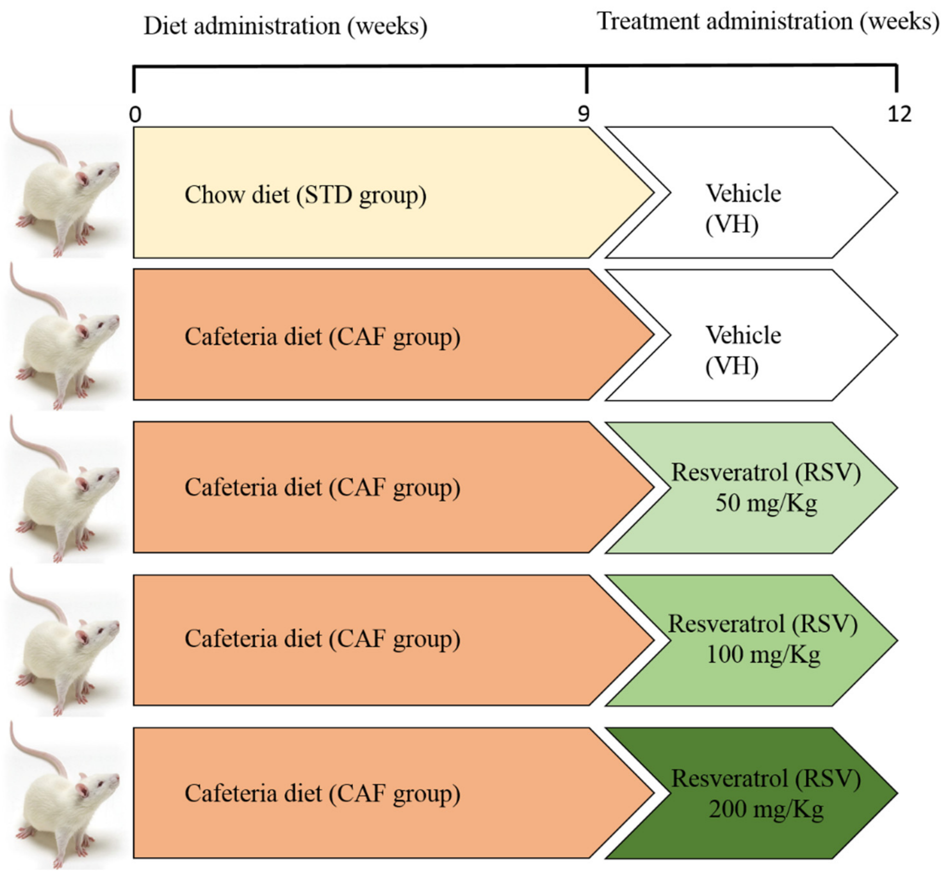

Figure S1. A scheme of the distribution of animals in the study. Each group was composed of 6 rats. During the first nine weeks, one group was fed the standard chow diet (STD group), whereas the other group was fed the cafeteria diet (CAF group). After nine weeks, the animals were orally administered either vehicle (VH) or resveratrol (RSV) + VH at three doses (50, 100 and 200 mg/kg). On week twelve, the animals were sacrificed. CAF: cafeteria diet; RSV: resveratrol; STD: standard chow diet; VH: vehicle.

Table S1. A summary of the rat-specific primer sequences used for qRT-PCR analysis.

Primer name	Direction	Primer sequences (5'-3')	Primer length (nucleotides)	Tm (ºC)	Amplicon lenght (nucleotides)
Acc	FW	gcggctctggaggtatatgt	20	51	156
	RV	tctgtttagcgtggggatgt	20	52	
Atf4	FW	attcttgcagcctcttccct	20	52	213
	RV	aggtaggactcagggctcat	20	49	
Chop	FW	tactcttgaccctgcatccc	20	51	170
	RV	actgaccactctgtttccgt	20	48	
Cpt1b	FW	tcatgtatcgccgcaaactg	20	55	199
	RV	agccaaaccttgaagaagcg	20	54	
Fas	FW	tggtgatagccggtatgtcc	20	52	153
	RV	tcagctttccagaccgctta	20	53	
iNos	FW	gtaccctcagttctgtgcct	20	48	220
	RV	tgttgcgttggaagtgtagc	20	51	
Pgc1a	FW	gtggatgaagacggattgcc	20	54	219
	RV	ggtgtggtttgcatggttct	20	52	
Ppara	FW	aacggcgttgaaaacaagga	20	55	191
	RV	aaggaggacagcatcgtgaa	20	52	
Ppia	FW	cttcgagctgtttgcagacaa	21	53	138
	RV	aagtcaccaccctggcacatg	21	57	
Ptp1b	FW	cccttttgaccacagtcgga	20	55	119
	RV	ttggtaaagggccctgggtg	20	58	
Scd1	FW	tgggttgccagtttctttcg	20	55	192
	RV	accacaagaagccacgtttc	20	52	
Socs3	FW	ctggacccattcgggagttc	20	56	148
	RV	ctgggagctaccgaccattg	20	54	
Ucp2	FW	agaccattgcacgagagaa	20	52	156
	RV	aagggaggtcgtcgttcatg	20	51	
Ucp3	FW	acgccattgtcaattgtgct	20	53	179
	RV	agcgttcatgtatcgggtct	20	51	
sXbp1	FW	ttaaggacacgcttggggat	20	54	193
	RV	gcaacagcgtcagaatccat	20	52	

Abbreviations: *Acc* (acetyl-CoA carboxylase), *Atf4* (activating transcription factor 4), *Chop* (DNA damage inducible transcript 3), *Cpt1b* (carnitine palmitoyltransferase 1b), *Fas* (fatty acid synthase), *iNos* (inducible nitric oxide synthase), *Pgc1a* (peroxisome proliferator-activated receptor gamma coactivator 1-alpha), *Ppara* (peroxisome proliferator activated receptor alpha), *Ppia* (peptidylprolyl isomerase a), *Ptp1b* (protein-tyrosine phosphatase 1b), *Scd1* (stearoyl-CoA desaturase 1), *Socs3* (suppressor of cytokine signalling 3), *Ucp2* (mitochondrial uncoupling protein 2), *Ucp3* (mitochondrial uncoupling protein 3), s*Xbp1* (spliced x-box binding protein 1).

Table S2. Chromatographic and fragmentation characteristics of RSV metabolites identified by UHPLC-MS^n in serum samples.

Metabolite	Abbreviation	RT (min)	[M-H]$^-$ (*m/z*)	MS^2 ions (*m/z*)	MS^3 ions (*m/z*)	MS^4 ions (*m/z*)
trans-resveratrol	RSV	2.89	**227**	185, 183, 159, 157, 143, 141		
resveratrol-4'-sulfate	R4S	2.30	**307**	*227*, 261	185, 183, 159, 157	
resveratrol-3-sulfate [a]	R3S	3.45	**307**	*227*, 243	185, 183, 159, 157	
resveratrol-4'-glucuronide [b]	R4G	2.57	**403**	*175, 227*	175: 113; 227: 185, 183, 159, 157	
resveratrol-3-glucuronide	R3G	2.75	**403**	*175, 227*	175: 113; 227: 185, 183, 159, 157	
resveratrol-disulfate [a]	RDS	3.89	**387**	*307*, 369	*227*	185, 183, 159, 157
resveratrol-glucuronide-sulfate [b]	RSG	2.25	**483**	*307*, 403, 227	*227*	185, 183, 159, 157
resveratrol-diglucuronide [b]	RDG	1.31	**579**	403		
dihydroresveratrol	DRSV	3.29	**229**	123		
dihydroresveratrol-sulfate [c]	DRS	2.84	**309**	*229*	123	
dihydroresveratrol-glucuronide [c]	DRG	2.24	**405**	175, *229*	123	
dihydroresveratrol-glucuronide-sulfate [c]	DRSG	2.25	**485**	*309*, 405	*229*	123, 187

MS^2 and MS^3 ions in italic were those subjected to MS^3 and MS^4 fragmentation for unambiguous identification. [a] Quantified as R4S equivalents [b] Quantified as R3G equivalents [c] Quantified as DRSV equivalents.

Table S3. Biochemical parameters of liver, calf skeletal muscle and eWAT.

	STD	CAF	CAF + RSV 50 mg/Kg	CAF + RSV 100 mg/Kg	CAF + RSV 200 mg/Kg
**Liver**					
Weight (%)	2.92 ± 0.12	2.94 ± 0.07	3.16 ± 0.04	3.11 ± 0.13	3.18 ± 0.07
TAG (mg/g)	6.81 ± 1.20	6.80 ± 0.59	5.31 ± 0.33	10.92 ± 0.72 $^{\#}$	5.25 ± 0.67
TC (mg/g)	1.85 ± 0.04	3.28 ± 0.33 *	2.38 ± 0.26 $^{\varepsilon}$	3.76 ± 0.25 $^{\#}$	2.66 ± 0.29 $^{\varepsilon}$
**Skeletal muscle**					
Weight (%)	0.60 ± 0.01	0.45 ± 0.01 *	0.44 ± 0.03	0.47 ± 0.01	0.51 ± 0.02
TAG (mg/g)	0.12 ± 0.01	0.12 ± 0.01	0.08 ± 0.02 $^{\varepsilon}$	0.03 ± 0.01 $^{\#}$	0.04 ± 0.01 $^{\#}$
TC (mg/g)	0.49 ± 0.06	0.54 ± 0.05 $^{\phi}$	0.25 ± 0.05 $^{\#}$	0.22 ± 0.06 $^{\#}$	0.24 ± 0.04 $^{\#}$
**eWAT**					
Weight (%)	2.04 ± 0.16	3.97 ± 0.27 *	4.07 ± 0.35	3.42 ± 0.16	2.91 ± 0.21 $^{\#}$
TAG (mg/g)	2.38 ± 0.09	2.56 ± 0.10	2.57 ± 0.14	3.04 ± 0.15 $^{\#}$	2.57 ± 0.11
TC (mg/g)	0.47 ± 0.05	0.67 ± 0.07 $^{\phi}$	0.82 ± 0.12	0.89 ± 0.15	0.50 ± 0.07

Data are expressed as a mean ± SEM, n=6. * $p < 0.05$ and $^{\phi}$ $p < 0.1$, T-student comparing CAF group respect to STD group. $^{\#}$ $p < 0.05$ and $^{\varepsilon}$ $p < 0.1$, T-student comparing RSV groups respect to CAF group. Abbreviations: CAF: cafeteria diet; eWAT: epididymal white adipose tissue; RSV: resveratrol; STD: standard chow diet; TAG: triacylglycerol; TC: total cholesterol.

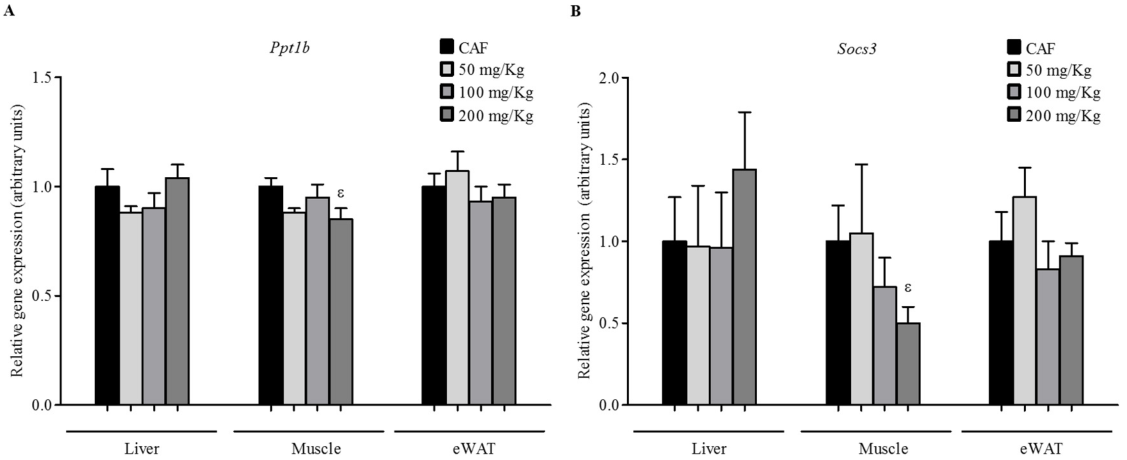

Figure S2. Inhibitors of the leptin signaling. The rats were fed the STD or CAF diet for 9 weeks. Then, the STD and CAF rats were treated orally with RSV (50, 100 or 200 mg per kg of body wt) or vehicle for 3 weeks. (A) Ptp1b gene expression; (B) Socs3 gene expression. Data are expressed as the mean ± SEM, n=6. $^{\#}$ $p < 0.05$ and $^{\varepsilon}$ $p < 0.1$, Student's t-test comparing the RSV group with the CAF group. CAF: cafeteria diet; eWAT: epididymal white adipose tissue; RSV: resveratrol; VH: vehicle; wt: weight. Ptp1b (protein-tyrosine phosphatase 1b); Socs3 (suppressor of cytokine signaling 3).

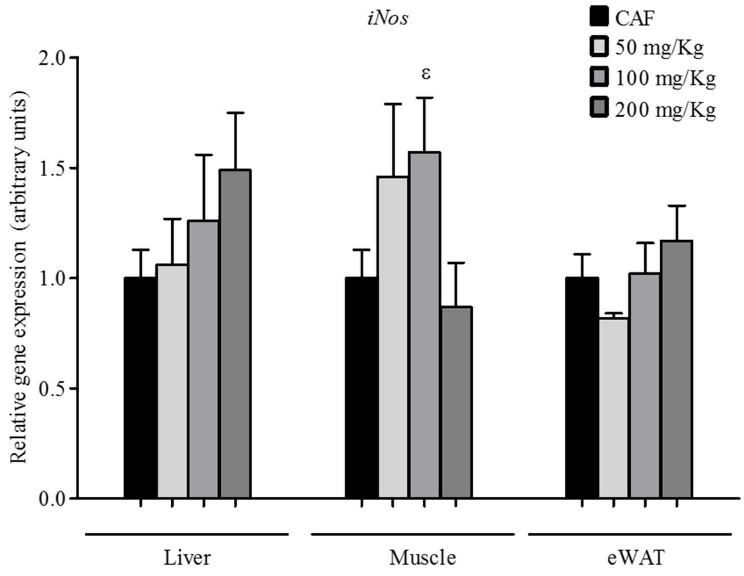

Figure S3. iNos gene expression**.** The rats were fed the STD or CAF diet for 9 weeks. Then, the STD and CAF rats were treated orally with RSV (50, 100 or 200 mg per kg of body wt) or vehicle for 3 weeks. Data are expressed as a mean ± SEM, n=6. $^{\#}p < 0.05$ and $^{\varepsilon}p < 0.1$, Student's t-test comparing the RSV group with the CAF group. CAF: cafeteria diet; eWAT: epididymal white adipose tissue; RSV: resveratrol; *iNos* (inducible nitric oxide synthase).

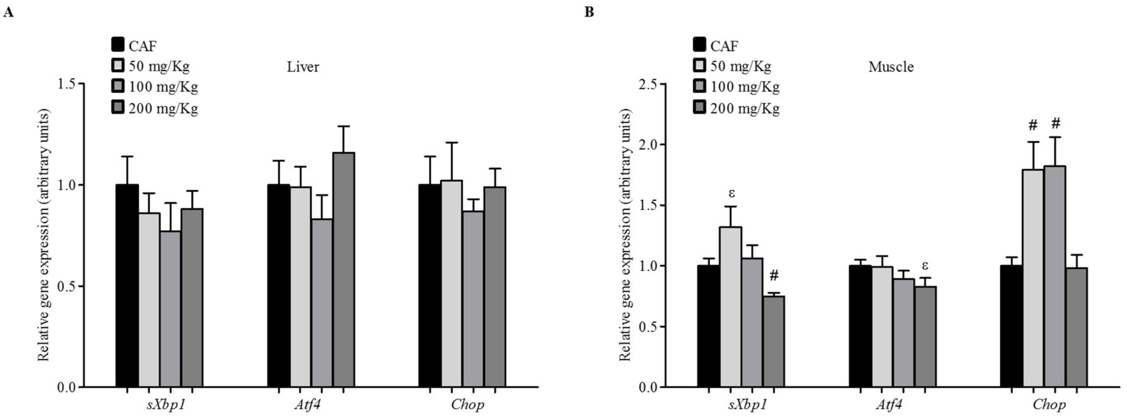

Figure S4. Gene expression of ER-stress markers. The rats were fed the STD or CAF diet for 9 weeks. Then, the STD and CAF rats were treated orally with RSV (50, 100 or 200 mg per kg of body wt) or vehicle for 3 weeks. (A) liver and (B) skeletal muscle.. Data are expressed as the mean ± SEM, n=6. $^{\#}$ $p < 0.05$ and $^{\varepsilon}$ $p < 0.1$, Student's t-test comparing the RSV group with the CAF group. CAF: cafeteria diet; RSV: resveratrol; Atf4 (activating transcription factor 4), Chop (DNA damage inducible transcript 3), sXbp1 (spliced x-box binding protein 1).

Table S4. Correlation analysis of the most relevant biochemical parameters and the metabolites concentrations of RSV present in the serum of rats treated with 50, 100 or 200 mg/kg RSV.

RSV Metabolites (µM)		Body wt gain (g)	Total body fat content (%)	Serum leptin (ng/mL)	LSI Liver	LSI Muscle	LSI eWAT
Phase II	R4G	ns	-0.67 *	-0.66 *	0.72 *	0.69 *	0.79 *
	R3G	ns	ns	ns	ns	ns	ns
	RDG	ns	ns	ns	0.60 #	ns	ns
	R4S	ns	-0.60 #	ns	ns	0.81*	0.79 *
	R3S	ns	-0.76 *	-0.60 #	ns	ns	ns
	RDS	ns	ns	ns	ns	ns	0.67 #
	RSG	ns	ns	ns	ns	0.67 #	ns
Microbiota	DRG	ns	ns	ns	0.60 #	ns	ns
	DRS	ns	ns	ns	ns	0.69 *	ns
	DRSG	-0.66 *	ns	ns	ns	0.81 *	0.71 *

Data are expressed as a mean ± SEM, n=6. Non-parametric Spearman test. * $p > 0.05$ and # $p > 0.1$. Abbreviations: eWAT: epididymal white adipose tissue; ns: non-significant; RSV: resveratrol; R4G: resveratrol-4'-glucuronide; R3G: resveratrol-3-glucuronide; R3S: resveratrol-3-sulfate; R4S: resveratrol-4'-sulfate; RDS: resveratrol-disulfate; RDG: resveratrol-diglucuronide; RSG: resveratrol-sulfate-glucuronide; DRG: dihydroresveratrol-glucuronide; DRS: dihydroresveratrol-sulfate; DRSG: dihydroresveratrol-sulfate-glucuronide.
